# Action-factorized Rainbow deep Q-network with token Transformer for computation offloading in edge computing-enabled Internet of Ships

**DOI:** 10.1371/journal.pone.0348376

**Published:** 2026-05-11

**Authors:** Shengtian Zhang, Haolin Yang, Hyeonseok Kim, Incheol Shin, Robert M. X. Wu

**Affiliations:** 1 Department of Artificial Intelligence Convergence, Pukyong National University, Busan, South Korea; 2 School of Computer and Network Engineering, Shanxi Datong University, Datong, China; 3 Faculty of Engineering and Information Technology, University of Technology Sydney, Sydney, Australia; Xidian University, CHINA

## Abstract

Edge computing (EC) in the Internet of Ships (IoS) reduces the latency and energy burdens of cloud-centric architectures, but fully realizing its benefits requires effective computation offloading strategies. Designing such strategies in dynamic maritime environments remains challenging due to the high-dimensional, combinatorial decision space, strict system constraints, and rapidly varying maritime wireless channels. This study proposes the action-factorized Rainbow deep Q-network (DQN) with token Transformer, a deep reinforcement learning (DRL) algorithm for discovering effective computation offloading strategies in EC-enabled IoS (EC-IoS). The core innovation of the algorithm lies in a novel action factorization mechanism coupled with our custom token Transformer-based state and action encoders, which effectively handle the complex decision space. Built upon Rainbow DQN and further accelerated with a parallel training architecture, the algorithm improves learning efficiency and stability. Experimental results illustrate that the computation offloading strategies learned by our algorithm significantly outperform multiple baselines on the weighted latency–energy objective. More importantly, these strategies achieve a zero rate of invalid actions, satisfy all system constraints, and ensure practical feasibility. Overall, the study demonstrates that the algorithm provides a robust method for computation offloading, effectively balancing latency and energy consumption in EC-IoS, thereby supporting maritime digitalization and automation.

## 1. Introduction

The Internet of Ships (IoS) is becoming increasingly important to maritime digitalization as the maritime industry continues to intensify. By linking ships, ports, shore-based infrastructure, and cloud services through heterogeneous networks and onboard sensors, IoS enables dynamic data sharing and cooperative decision-making in maritime environments. This has enabled a wide range of intelligent applications, including autonomous navigation, cargo tracking, remote maintenance, and smart port operations [1–3]. However, the traditional IoS architecture relies mainly on cloud computing frameworks for data processing and service delivery. Cloud computing frameworks often fail to meet the latency, reliability, and privacy requirements of maritime scenarios, especially for intelligent ships while navigating. In addition, the traditional IoS architecture leads to high energy consumption and places a heavy burden on the network infrastructure.

To alleviate the limitations of cloud computing frameworks, edge computing (EC) has been introduced into maritime environments to improve IoS efficiency. EC, which is a new computing paradigm, represents a distributed extension of cloud computing toward the network edge. It aims to offload user tasks to edge servers, which helps reduce the load on centralized cloud datacenters and simultaneously lowers the latency and energy consumption of terminal devices [4,5]. In IoS, shore-based infrastructure, smart buoys, and unmanned aerial vehicles (UAVs) can all work as edge servers to provide computing and storage to nearby ships.

Although EC provides advantages in reducing latency and improving resource utilization, the resource-constrained maritime environment hinders its effectiveness. Edge servers deployed in EC-enabled IoS (EC-IoS) are often limited in computational capacity, energy supply, and network stability. Therefore, efficiently distributing computational tasks among local ships, EC nodes, and cloud data centers, commonly referred to as computation offloading, has become a critical problem. Effective computation offloading strategies can address several challenges, including varying task workloads, diverse network conditions, energy limitations, and latency requirements [6,7]. Developing a suitable computation offloading algorithm is essential for leveraging the potential of EC-IoS, and recent studies have proposed various methods to this end. Yang et al. [8] proposed a lightweight multi-armed bandit (MAB) learning framework for selecting edge servers in maritime edge intelligence networks. They also designed the UCB1-ESSS algorithm to optimize the balance between the latency and energy consumption of computation offloading. Dai et al. [9] researched a mobile edge computing (MEC) framework that combines offshore and aerial multi-access, providing a means for unmanned surface vehicles (USVs) to offload computational tasks to both offshore base stations and UAVs using the frequency division multiple access (FDMA) and non-orthogonal multiple access (NOMA) schemes simultaneously. Their optimized approach demonstrated reductions in task latency and improved channel utilization in complex marine topological structures. Although the studies aforementioned have shown significant progress, the corresponding algorithms are relatively complex and lack the integration of intelligent optimization methods, such as deep reinforcement learning (DRL), which limits their scalability and generalizability. Meanwhile, research on computation offloading in EC-IoS remains relatively sparse, and effective methods for dealing with resource-constrained and dynamic maritime scenarios still require further investigation.

To address the challenges above, this study proposes a DRL algorithm, action-factorized Rainbow deep Q-network with token Transformer (AF-TT-RDQN), for optimizing computation offloading in EC-IoS. The objective is to obtain optimal strategies that minimize the weighted sum of the latency and energy consumption within the system while also meeting the relevant constraints. The main contributions of this study are summarized as follows:

1) We develop a system model for the EC-IoS environment, taking into account the heterogeneous nature of ships and EC nodes. Based on the system model, we formulate the complex and dynamic computation offloading problem as a Markov decision process (MDP), capturing the temporal dependencies and stochastic characteristics of the EC-IoS, including time-varying channel conditions, dynamic task arrivals, and fluctuating edge resource utilization.
2) Based on the MDP, to effectively address the high-dimensional, combinatorial decision space in offloading tasks that involve multiple ships with multinode and multichannel contexts, we propose AF-TT-RDQN, which incorporates an action factorization mechanism and token Transformer modules to represent states and actions concisely. Built upon the Rainbow deep Q-network (DQN), AF-TT-RDQN systematically incorporates six advanced components: Double DQN, Dueling Network, prioritized experience replay (PER), distributional DQN, multi-step learning, and NoisyNet. A parallel training framework is also utilized to increase learning stability and reduce overall runtime.
3) We conduct experiments to validate the effectiveness of computation offloading strategies learned by the proposed algorithm. The results demonstrate its superiority in solving the defined computation offloading problem over several baseline methods, including other DRL algorithms, such as Advantage Actor–Critic (A2C) [10], Trust Region Policy Optimization (TRPO) [11], and Proximal Policy Optimization (PPO) [12]. In particular, the computation offloading strategies learned by AF-TT-RDQN achieve the highest total reward of −105.83. It outperforms PPO by 6.7%, TRPO by 15.5%, and A2C by 22.8%. The strategies also maintain an improved balance between latency and energy consumption, while satisfying all system constraints. Given the effectiveness of AF-TT-RDQN, it can provide underlying technical support for EC-IoS, promoting the development of maritime digitalization and automation.

The rest of this paper is organized as follows. Related works are reviewed in Section 2. Section 3 describes the system model for computation offloading and problem formulation. In Section 4, the proposed algorithm is presented. Section 5 presents the experiments and analyzes the results of the performance evaluation. Finally, Section 6 concludes the study and discusses potential directions for future work.

## 2. Related works

### 2.1 EC-IoS

IoS is an extension of the Internet of Things (IoT) in maritime environments. It is an integrated network system that utilizes sensors, heterogeneous networks, and communication technologies to connect various marine aspects, including ships, ports, equipment, and environmental facilities, for data collection, exchange, and processing. Applications for IoS include ship condition monitoring, route planning, and port scheduling [3,13,14]. Some illustrative examples of IoS in the maritime industry include the e-Navigation system developed by the International Maritime Organization (IMO), the River Information System platform in Europe, and the Ship Area Network in South Korea. Although IoS is promising in unleashing the potential for intelligent maritime operations, some fundamental challenges still exist in the current stage. For instance, the cloud computing frameworks of IoS mainly focus on information exchange without offering effective mechanisms for real-time collaborative processing. Additionally, the high susceptibility of maritime communication networks to environmental interferences often results in unstable and fluctuating connections with delays [15]. Therefore, research on employing EC and intelligent optimization algorithms improving the reliability and performance of the IoS can be a promising direction.

EC is a new computing paradigm that emphasizes low latency and high-reliability services providing strong support for IoS. Wang et al. [16] proposed a joint optimization strategy for computation offloading and resource allocation in maritime MEC systems, utilizing EC with coastal base stations (CBSs) and maritime information stations (MISs). Zhang et al. [17] used UAVs equipped with Intelligent Reflecting Surfaces (IRSs) to provide communication and computation services, enabling EC-IoS. These methods, based on EC, have significantly reduced the data transmission paths with the IoS, thereby decreasing the task response time and reducing energy consumption. In a traditional cloud computing framework, however, due to the limited bandwidth associated with the network and the transmission time, it cannot safely address task latency, which affects critical maritime tasks such as navigation safety, collision avoidance, and route planning. Increased energy consumption also remains an issue related to the traditional framework. Additionally, EC offers more flexible resource scheduling in IoS. Its distributed characteristics provide robust support for scaling and fault tolerance of the system.

EC-IoS has emerged and continues to be established as a viable technology that supports the push for maritime development. At present, collaboration between industry and academia is underway. Currently, as they collaborate, EC-IoS is in a phase of practical deployment. However, the current communication and energy conditions associated with open sea navigation scenarios present significant challenges in the effective usage of EC-IoS. Some studies have mentioned that UAVs could be deployed to facilitate EC services; however, UAV systems are typically expensive to deploy and maintain, so their potential application would be limited to specific situations. Therefore, this study focuses on near-shore environments, where EC resources are provisioned through shore-based infrastructure and smart buoys.

### 2.2 Intelligent computation offloading technology

To maximize the potential of EC-IoS in maritime scenarios, it is essential to design intelligent computation offloading strategies. Under EC-IoS, computation offloading is a process where a ship offloads part or all of its local computing tasks to edge servers or cloud servers that compute the tasks and return the computed results to the ship. This considerably reduces system latency and the energy consumption of devices, thereby minimizing the overall costs of the system [18]. Compared with terrestrial communications, EC-IoS has the characteristics of more dynamic network conditions and limited computing resources, which significantly increases the complexity of making efficient computation offloading strategies.

Several studies have applied intelligent computation offloading to maritime network environments. Yang et al. [19] designed a multivessel computation offloading algorithm based on an improved Hungarian method to minimize the energy consumption and task latency in maritime networks. Jiang et al. [20] proposed an improved Grey Wolf Optimization algorithm, which is used to implement the offloading of computing tasks in a maritime EC-based network, effectively reducing energy consumption while satisfying the latency requirements. The studies mentioned above demonstrated exemplary performance in computation offloading in maritime environments. However, these algorithms exhibit coarse-grained scheduling, and their global convergence and solution optimality cannot be guaranteed. Consequently, they are prone to becoming stuck in local optimal solutions. Several related studies employ a multistage or multitier method of computation offloading, which can lead to more efficient decision-making than traditional or heuristic algorithms when applied to dynamic and resource-constrained marine environments. Yang et al. [21] employed a two-stage offloading optimization algorithm to address the energy–latency tradeoff in maritime IoT, which jointly optimizes communication and computation resource allocation to minimize the total system cost efficiently. Dai et al. [22] proposed a two-tier computation offloading scheme for marine edge computing networks based on incentives. The use of a hybrid Stackelberg auction game-based method of incentives in a two-tier architecture effectively improved offloading efficiency and equipment utilization. Although these approaches have demonstrated effective computation offloading optimization, the algorithms exhibit high complexity and have limitations in scalability and generalizability. By incorporating artificial intelligence technologies such as DRL, the above problems can be alleviated. DRL incorporates the powerful perception and generalization capabilities of deep learning to process high-dimensional and complex information, thereby enhancing the capability of reinforcement learning in making the best possible long-term decisions in a complex environment [23–25]. DRL has demonstrated significant effectiveness and high applicability in various road transportation scenarios, including computation offloading and resource allocation in the Internet of Vehicles (IoV) [26–29]. Specifically, in IoV networks, high-speed mobility can induce rapidly varying network topologies and uncertain transmission conditions, causing challenges for scheduling, optimization, and decision-making [30]. To deal with such highly dynamic environments, Xie et al. [31] developed a DRL-based computation offloading framework for space–air–ground integrated vehicle networks, leveraging DQN-style learning and improved experience replay to improve learning efficiently and offloading performance. In maritime environments, some studies have also applied DRL to areas like autonomous ship navigation, route planning, and collision avoidance [32,33]. However, few studies have used DRL algorithms to solve computation offloading optimization problems in EC-IoS scenarios. Therefore, this study proposes the use of an improved DRL algorithm, AF-TT-RDQN, to optimize computation offloading in EC-IoS, aiming to minimize system latency and energy consumption efficiently.

## 3. System model and problem formulation

### 3.1 System model

**Fig 1:**
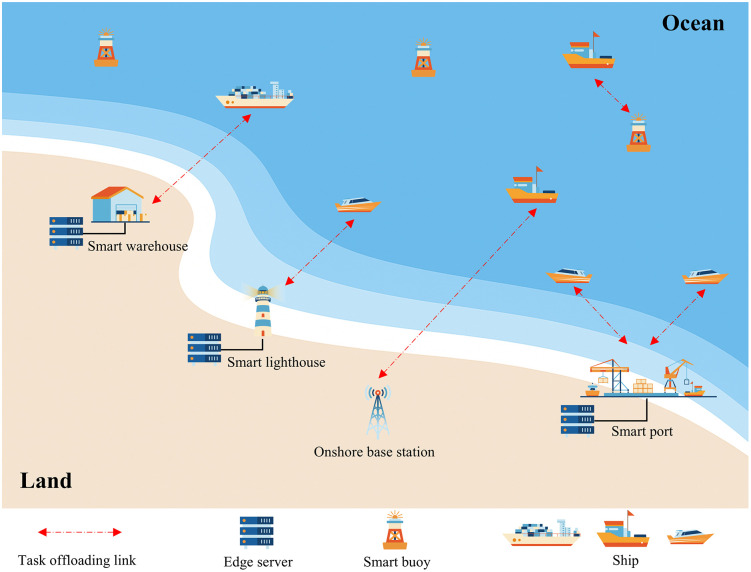
EC-IoS system model.

The system model for the EC-IoS scenario is illustrated in Fig 1. This study mainly focuses on the computation offloading problem between ships and EC nodes in near-shore environments. It can be seen from Fig 1 that some shore-based infrastructure equipped with servers, such as ports, warehouses, lighthouses, and onshore base stations, can serve as EC nodes. At sea, smart buoys also function as EC nodes. All of these entities provide EC resources, with their computing capabilities varying according to type. Similarly, ships have heterogeneous computational requirements depending on their categories. In the system model, EC nodes are assumed to be stationary. For tractability, ship locations are modeled as quasi-static over the considered time horizon. Therefore, the ship-node distances are time-invariant. This modeling choice is consistent with prior maritime offloading works that treat infrastructure locations as fixed and adopt a quasi-static communication geometry [34,35]. As shown in Fig 1, ships located farther from the coast can only offload their tasks to smart buoys owing to long communication distances. In contrast, ships operating closer to the shore have access to a broader range of offloading options, enabling them to choose between nearby nodes and more powerful shore-based infrastructure with higher computing capacity. Determining the optimal choice in such scenarios requires an intelligent computation offloading algorithm.

In the system model, the ship index is denoted as i, where i∈{1,2,...,M}. Each ship can generate one or more computation tasks. These tasks are independent of each other, and task dependencies are not considered during the offloading process. Let j represents the j th EC node, where j∈{1,2,...,N}. Each ship is allowed to offload tasks to only one designated EC node, whereas an EC node can simultaneously handle computation tasks from multiple ships. By adopting effective computation offloading strategies, task execution latency in the system can be significantly reduced, and the energy consumption of ships can be minimized.

### 3.2 Computing offloading cost model

According to the system model mentioned above, computation tasks can either be executed locally on a ship or offloaded to an EC node. When a computation task is executed locally, the time required for this task to be executed is:


$$\overset{local}{\underset{i}{T}}=\frac{c_{i}}{f_{i}},$$ (1)

where $c_{i}$ represents the number of CPU cycles required to execute each computation task on the ship i, and $f_{i}$ denotes the local computing frequency of the ship i.

To prevent the execution latency of a computation task from exceeding its maximum allowable latency $\overset{max}{\underset{i}{T}}$, the task can also be offloaded to an appropriate EC node. The latency of a computation task offloaded to an EC node consists of four components: uplink transmission delay, queuing delay at the EC node, computation processing delay, and downlink transmission delay for returning the computation result. Because the downlink bandwidth is typically large, the data transmission rate is relatively high, and the amount of data returned after computation is comparatively small, the impact of downlink transmission can be neglected [36]. The total offloading latency is obtained as follows:


$$\overset{edge}{\underset{i,j}{T}}=\overset{up}{\underset{i,j}{T}}+\overset{wait}{\underset{i,j}{T}}+\overset{exe}{\underset{i,j}{T}}.$$ (2)

We assumed that each EC node has K orthogonal subchannels, and the bandwidth of each subchannel is W. According to the Shannon capacity theorem, the uplink transmission rate for the offloaded task from the ship i to the EC node j is given by the following channel model [16]:


$$r_{i,j}=X_{i,j}\sum_{k=0}^{K-1}\overset{k}{\underset{i,j}{Y}}W\;{log}_{\text{2}}(1+\frac{\overset{k}{\underset{i,j}{p}}\overset{k}{\underset{i,j}{\beta }}{\left|\overset{k}{\underset{i,j}{h}}\right|}^{\text{2}}}{\sigma^{\text{2}}}),$$ (3)

where the binary indicator $X_{i,j}$ denotes an offloading decision. If $X_{i,j}=1$, the ship i offloads its computation tasks to the EC node j for execution; otherwise, $X_{i,j}=0$. The binary indicator $\overset{k}{\underset{i,j}{Y}}$ represents the subchannel allocation decision, which is equal to 1 if the subchannel k of the EC node j is allocated to the ship i, and 0 otherwise. The transmission power of the ship i on the channel to the EC node j is set to the value $\overset{k}{\underset{i,j}{p}}$. $\sigma^{\text{2}}$ is the noise power. $\overset{k}{\underset{i,j}{\beta }}$ represents the large-scale path loss gain coefficient on the k th subchannel, which is expressed as:


$$\overset{k}{\underset{i,j}{\beta }}={\left(\frac{\lambda_{j,k}}{4\pi d_{i,j}}\right)}^{\text{2}},$$ (4)

where $\lambda_{j,k}$ denotes the wavelength of the k th subchannel of EC node j, and $d_{i,j}$ is the distance between the ship i and EC node j. Equation (4) omits the height-dependent interference term, which is negligible in near-shore deployments with relatively stable antenna height. $\overset{k}{\underset{i,j}{h}}$ is the small-scale fading coefficient of the ship i to the EC node j on the k th subchannel. Considering the strong line of sight (LOS) component in near-shore environments, along with reflections from the water surface, waves, and coastline, the channel characteristics are modeled using a Rician fading model:


$$\overset{k}{\underset{i,j}{h}}=\sqrt{\frac{K_{r}}{K_{r}+1}}+\sqrt{\frac{\text{1}}{K_{r}+1}}\overset{k}{\underset{i,j}{s}},$$ (5)

where $K_{r}$ is the Rician factor and $\overset{k}{\underset{i,j}{s}}$ follows a complex Gaussian distribution CN(0,1).

According to the uplink transmission rate $r_{i,j}$, the uplink transmission delay $\overset{up}{\underset{i,j}{T}}$ can be calculated as follows:


$$\overset{up}{\underset{i,j}{T}}=\frac{b_{i}}{r_{i,j}},$$ (6)

where $b_{i}$ denotes the input data size of each computation task on the ship i. It is assumed that each EC node can time-share its total computing capacity among several tasks. Based on this, the computation processing delay of an offloaded task at the EC node j is given by:


$$\overset{exe}{\underset{i,j}{T}}=\frac{c_{i}\overset{exe}{\underset{j}{n}}}{f_{j}},$$ (7)

where $\overset{exe}{\underset{j}{n}}$ denotes the number of tasks concurrently executed at EC node j, and $f_{j}$ denotes the computing frequency of EC node j. The overall frequency $f_{j}$ is evenly sliced and allocated to the tasks being served concurrently. The waiting time in the task queue at the EC node j is then estimated using the M/M/1 queuing model, which assumes Poisson arrivals and exponential service times. We adopt the M/M/1 queuing model to obtain a tractable and reproducible estimate of the average queuing delay at each EC node. Specifically, instead of explicitly tracking the queue length evolution as additional system states, we characterize the queue at EC node j by its aggregate task arrival rate and the effective service rate. This abstraction yields a closed-form delay estimate and allows us to enforce queue stability. Assuming equal queuing delays among tasks, we approximate $\overset{wait}{\underset{i,j}{T}}\approx \overset{wait}{\underset{j}{T}}$. The queuing delay at the EC node is then given by:


$$\overset{wait}{\underset{i,j}{T}}=\frac{g_{j}}{u_{j}(u_{j}-g_{j})},$$ (8)

where $g_{j}$ denotes the aggregate arrival rate of the computation tasks offloaded to EC node j. $g_{j}$ is obtained as:


$$g_{j}=\sum_{i=1}^{M}X_{i,j}g_{i}.$$ (9)

$g_{i}$ denotes the task arrival rate of the ship i. The service rate of an EC node j reflects its capability to process offloaded computation tasks and is given by:


$$\mu_{j}=\frac{f_{j}}{\overset{―}{c_{j}}},$$ (10)

where ${\overset{―}{c}}_{j}$ is the average computational complexity of the tasks that are executed in parallel on the EC node j at the current time slot.

According to the above formulas, the overall system latency is given as follows:


$$T=\sum_{i=1}^{M}g_{i}\left(\sum_{j=1}^{N}X_{i,j}\overset{edge}{\underset{i,j}{T}}+\left(1-\sum_{j=1}^{N}X_{i,j}\right)\overset{local}{\underset{i}{T}}\right).$$ (11)

The local energy consumption $\overset{local}{\underset{i}{E}}$ denotes the amount of energy required by a ship to execute a task locally when no offloading is performed. It is given by:


$$\overset{local}{\underset{i}{E}}=z_{i}\overset{\text{2}}{\underset{i}{f}}c_{i},$$ (12)

where $z_{i}$ denotes the ship-specific energy coefficient determined by the underlying hardware architecture.

This study mainly focuses on the energy optimization of ship-side operations. Therefore, when a computation task is offloaded, the corresponding energy consumption only considers the energy incurred during the uplink transmission process. $\overset{edge}{\underset{i,j}{E}}$ is expressed as:


$$\overset{edge}{\underset{i,j}{E}}=\left(X_{i,j}\sum_{k=0}^{K-1}\overset{k}{\underset{i,j}{Y}}\overset{k}{\underset{i,j}{p}}\right)\overset{up}{\underset{i,j}{T}}.$$ (13)

According to the above formulas, the overall energy consumption is defined as:


$$E=\sum_{i=1}^{M}g_{i}\left(\sum_{j=1}^{N}X_{i,j}\overset{edge}{\underset{i,j}{E}}+(1-\sum_{j=1}^{N}X_{i,j})\overset{local}{\underset{i}{E}}\right).$$ (14)

### 3.3 Problem formulation

Appropriate offloading strategies can effectively reduce the execution latency of computation tasks in the system as well as the energy consumption of ship-side operations. The optimization objective of this study is to minimize the weighted sum of latency and energy consumption for all computation tasks of ships in the system. Both latency and energy consumption are important in EC-enabled IoS, but their relative importance is scenario-dependent. Latency is typically more important for time-sensitive services, such as safety-related alerting, collision avoidance, and real-time situational awareness. In contrast, energy is often more important for energy-constrained maritime devices and platforms, such as battery-powered sensors or buoys, small unmanned platforms, and ships operating under strict power budgets. In this study, we can capture different operating priorities through the reward weights in the weighted-sum objective. By assigning a larger weight to latency or to energy, the same AF-TT-RDQN framework can be used to optimize latency-critical or energy-critical missions, respectively. However, since latency and energy consumption have different physical units and scales, directly summing them may cause a weight imbalance, leading to biased optimization results. To eliminate the influence of dimensional inconsistency, data normalization is required. Specifically, the min-max normalization method is adopted for this purpose:


$$\tilde{T}\left(X,Y\right)=\frac{T\left(X,Y\right)-T_{min}}{T_{max}-T_{min}},$$ (15)

where $X=\left\{X_{i,j}\right\}$ denotes the offloading decision matrix for computation tasks, and $Y=\left\{\overset{k}{\underset{i,j}{Y}}\right\}$ denotes the subchannel allocation matrix. T(X,Y) represents the system latency for a given computation offloading strategy. $T_{min}$ and $T_{max}$ denote the minimum and maximum latency, respectively.


$$\tilde{E}\left(X,Y\right)=\frac{E\left(X,Y\right)-E_{min}}{E_{max}-E_{min}}.$$ (16)

Similarly, E(X,Y) denotes the energy consumption under the given computation offloading strategy. $E_{min}$ and $E_{max}$ represent the minimum and maximum energy consumption values, respectively.

In this work, the min-max normalization bounds $T_{min}$, $T_{max}$, $E_{min}$, and $E_{max}$ are implemented as global constants shared across all time slots, rather than being recomputed per time slot. Specifically, the bounds are estimated offline prior to training by sampling a set of time-varying environment realizations using the same stochastic generators as in the simulation. For the relatively small-scale setting in this work, we approximate the global extrema by exhaustively enumerating feasible offloading strategies for each sampled realization, and then taking the global minima and maxima over all collected delay and energy values as the normalization bounds. We use $T_{min}=4$ s, $T_{max}=40$ s, $E_{min}=0.5$ J, and $E_{max}=50$ J, which are kept unchanged during both training and testing. For larger-scale settings where exhaustive evaluation becomes expensive, the global bounds can be obtained using Monte Carlo sampling. This is done by sampling time-varying environment realizations and evaluating a subset of feasible strategies, without modifying the underlying method.

Based on the above formulas, the latency and energy consumption are normalized to the range of [0,1] before applying the weighted sum method. The problem can then be formulated as follows:


$$P1:\underset{X,Y}{min}\alpha \tilde{T}\left(X,Y\right)+\beta \tilde{E}\left(X,Y\right),$$ (17)

s.t.


$$C1:\text{\textbackslash{}hspace\{0.17em}\}X_{i,j}\in \left\{0,1\right\},\overset{k}{\underset{i,j}{Y}}\in \left\{0,1\right\},\forall i,j,k,$$

$$C2:\text{\textbackslash{}hspace\{0.17em}\}\sum_{j=1}^{N}X_{i,j}\leq 1,\forall i,$$

$$C3:\text{\textbackslash{}hspace\{0.17em}\}\sum_{i=1}^{M}\overset{k}{\underset{i,j}{Y}}\leq 1,\forall j,k,$$

$$C4:\text{\textbackslash{}hspace\{0.17em}\}\overset{k}{\underset{i,j}{Y}}\leq X_{i,j},\forall i,j,k,$$

$$C5:\text{\textbackslash{}hspace\{0.17em}\}X_{i,j}\leq \sum_{k=1}^{K}\overset{K}{\underset{i,j}{Y}}\leq KX_{i,j},\forall i,j,$$

$$C6:\text{\textbackslash{}hspace\{0.17em}\}\sum_{j=1}^{N}\sum_{k=1}^{K}\overset{k}{\underset{i,j}{p}}\overset{k}{\underset{i,j}{Y}}\leq \overset{max}{\underset{i}{P}},\forall i,$$

$$C7:\text{\textbackslash{}hspace\{0.17em}\}g_{j}<u_{j},\forall j,$$

$$C8:\text{\textbackslash{}hspace\{0.17em}\}\overset{exe}{\underset{j}{n}}\leq \overset{max}{\underset{j}{N}},\forall j,$$

$$C9:\text{\textbackslash{}hspace\{0.17em}\}T_{i}\leq \overset{max}{\underset{i}{T}},$$

C10:\hspace{0.17em}α+β=1.

In equation (17), C1 defines the domain of the decision variable. C2 represents that each ship i can select at most one EC node j for offloading tasks; if all values are zero, tasks will be executed locally. C3 indicates that the subchannel can serve only one ship in the same time slot to avoid cross-channel interference. C4 and C5 guarantee consistency between the offloading decision and the subchannel allocation. If the task is not offloaded to a node j, no subchannel can be allocated to that ship. Conversely, if the task is offloaded, at least one subchannel must be assigned, with a maximum of K subchannels. C6 indicates that the total transmission power must not exceed the maximum power limit of the ship’s power $\overset{max}{\underset{i}{P}}$. C7 ensures the stability of the M/M/1 queue, thereby guaranteeing a finite waiting time. C8 represents the limitation on the number of processing threads at the EC node j. C9 states that the execution time $T_{i}$ of a ship’s tasks must not exceed the maximum allowable latency $\overset{max}{\underset{i}{T}}$. C10 indicates that the sum of the weight parameters for delay α and energy consumption β is equal to one.

According to equation (17), optimization problem P1 is a non-convex and mixed-integer nonlinear programming (MINLP) problem [37]. Due to its high-dimensional, combinatorial action space and intensive constraints, traditional optimization methods may suffer from low computational efficiency when applied to such complex problems. To address these challenges, we propose an algorithm for this class of optimization tasks.

## 4. Computation offloading algorithm

### 4.1 Markov decision process

Computation offloading based on reinforcement learning can be mathematically formulated as an MDP [38,39]. Within the MDP framework, the state of the agent evolves according to its actions, and the agent receives the corresponding rewards at each state transition. Through continuous interaction with the environment, the agent learns action strategies that maximize the cumulative reward. The key elements of the MDP for the computation offloading problem are defined as follows:

1) State: The state primarily includes information about the utilization of computation resources, network channel conditions, and the resource allocation status of EC nodes. Therefore, the characteristics of ships, EC nodes, and communication channels should all be incorporated into the state. The system state at each decision slot t is defined as:

$$\begin{array}{cc} s_{t}=\; & \{x_{i},y_{i},c_{i},b_{i,}f_{i},\overset{max}{\underset{i}{P}},z_{i},g_{i}\left(t\right),\overset{limit}{\underset{i}{T}}\left(t\right),\\ & \;x_{j},y_{j},f_{j},\overset{max}{\underset{j}{N}},\rho_{j}(t-1),\overset{―}{c_{j}}(t-1),\\ & \;\lambda_{j,k},\overset{k}{\underset{i,j}{h}}\left(t\right),\overset{k}{\underset{i,j}{p}}\},\\ & \;\forall i\in \{1,...,M\},\forall j\in \{1,...,N\},\forall k\in \{1,...K\}, \end{array}$$ (18)

where $x_{i}$ and $y_{i}$ represent the location coordinates of the ship i. $g_{i}\left(t\right)$ denotes the task arrival rate of ship i at slot t. $\overset{\text{limit}}{\underset{i}{T}}\left(t\right)$ denotes the maximum allowable latency for ship i in slot t. $x_{j}$ and $y_{j}$ represent the location coordinates of EC node j. $\overset{k}{\underset{i,j}{h}}\left(t\right)$ is the small-scale fading coefficient of ship i to EC node j on the k th subchannel at slot t. ${\overset{―}{c}}_{j}(t-1)$ is the average computational complexity of the tasks executed in parallel on EC node j at the previous slot t−1. $\rho_{j}(t-1)$ denotes the utilization of EC node j in the previous time slot t−1, and is given by:


$$\rho_{j}(t-1)=\frac{g_{j}(t-1)}{u_{j}(t-1)}.$$ (19)

2) Action: In each decision slot t, the action refers to the joint offloading decision made by all ships. Each ship can either execute tasks locally or offload tasks to a selected EC node and allocate one or more available subchannels for communication. The action at the slot t is defined as:

$$a_{t}=\left\{\left(EN_{i},CH_{i}\right)\right\},\forall i\in \{1,...,M\},$$ (20)

where $EN_{i}\in \{0,1,...,N\}$ is the selected EC node for ship i; $EN_{i}=0$ indicates that the task is executed locally; $CH_{i}\subseteq \{1,2,...,K\}$ represents the set of subchannels allocated to ship i when offloading is selected. If $EN_{i}=0$, then $CH_{i}=\varphi$.

3) Reward: The reward is designed to reflect the efficiency of computation offloading strategies with the problem formulation mentioned above. It is given by:

$$R_{t}=\{\begin{array}{c} -\left(\alpha \tilde{T}\left(X,Y)+\beta \tilde{E}(X,Y\right)\right),\text{if all constraints are satisfied}\\ -L_{penalty},\text{otherwise} \end{array}.$$ (21)

To ensure constraint satisfaction, the agent receives a large negative penalty for violating any constraints. This reward mechanism encourages the agent to learn computation offloading strategies that are not only latency-aware and energy-aware but also compliant with system constraints.

### 4.2 Proposed algorithm

**Fig 2:**
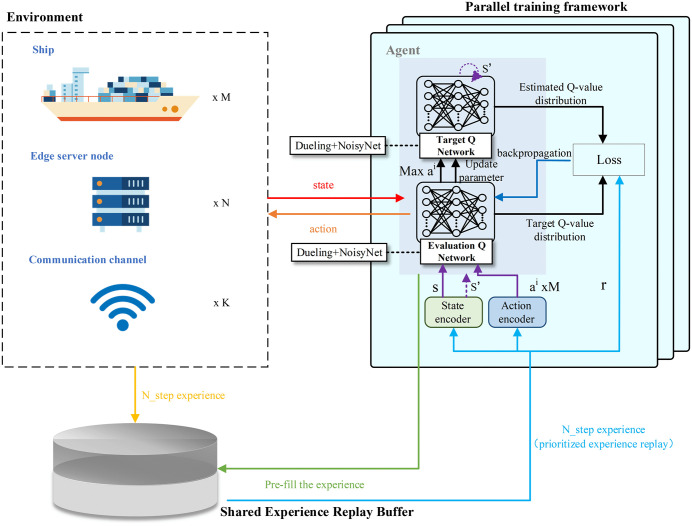
Training framework of AF-TT-RDQN.

Through the analysis of the MDP mentioned above, we designed AF-TT-RDQN by integrating action factorization, token Transformer-based state and action encoders, Rainbow DQN, and a parallel training mechanism. This algorithm effectively addresses the action space explosion problem arising from the triple combination of ships, EC nodes, and communication channels in EC-IoS scenarios. Relying on the excellent learning ability of the model, the algorithm can achieve efficient computation offloading strategies. The training framework of AF-TT-RDQN is illustrated in Fig 2. The rest of this section elaborates on the core components, key mechanisms, and operational flow of AF-TT-RDQN.

#### 4.2.1 Action factorization.

The action space considered in this study is large, discrete, and grows exponentially with the number of ships, EC nodes, and channels. Since the action space grows exponentially, we introduce the concept of action factorization inspired by Factorized Q and BDQ [40–42]. In the factorized Q function approach, the joint action space is decomposed into multiple subactions, each corresponding to a single ship. For each subaction, a local Q function is constructed, and these local Q functions are subsequently aggregated to obtain the global Q value:


$$Q\left(s,a\right)=\sum_{i=1}^{M}Q_{i}\left(s,a_{i}\right).$$ (22)

#### 4.2.2 Token transformer encoding.

This study adopts the state encoder and action encoder inspired by the Transformer to facilitate the aforementioned action factorization.

**Fig 3:**
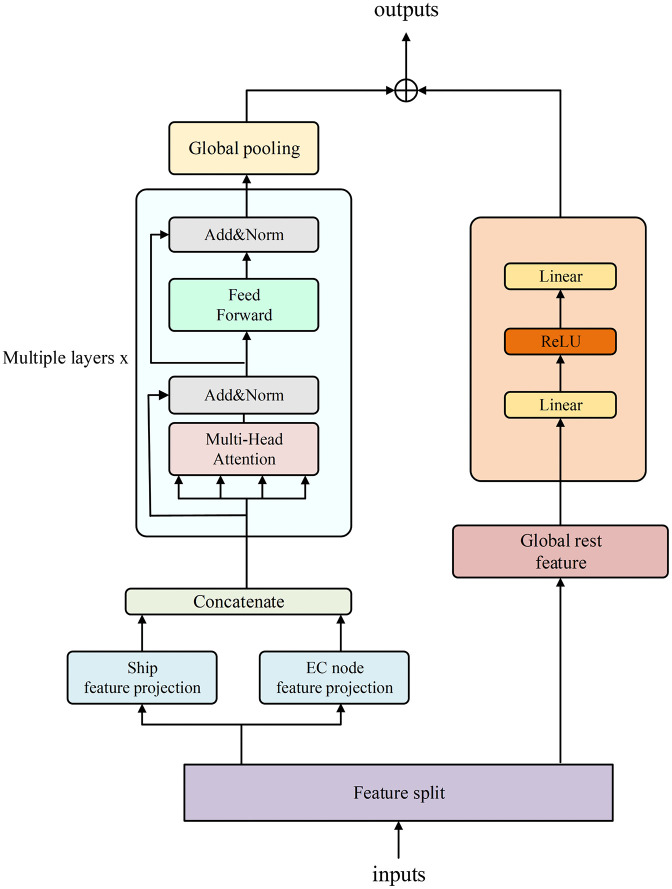
Architecture of the state encoder.


The architecture of the state encoder is illustrated in Fig 3. The input of the encoder is the system state vector s consisting of per ship features, per edge server features, and global physical features, such as the wavelength matrix, channel gains, and transmit power:

$$s\in R^{D}\;D=9M+6N+NK+2MNK.$$ (23)

Through the encoder, this high-dimensional input is compressed into a fixed-length embedding $\text{z}\in {\text{R}}^{d}\;$. To effectively and automatically capture the structured interactions among ships, between ships, and EC nodes, as well as among EC nodes themselves, from the high-dimensional state, the encoder first decomposes the state vector into three components: ship features, EC node features, and global physical features, as summarized in Table 1. The ship features and EC node features are independently projected into a unified dimension d via separate linear mappings, which facilitates the implementation of parameter sharing and multi-head attention calculations in the subsequent Transformer encoder. These two sets of features are then combined into a single unified token sequence and fed into the Transformer encoder. The Transformer encoder utilizes four attention heads and omits positional encoding when calculating self-attention over the token sequence, thereby facilitating effective information exchange among tokens. With the token embedding dimension set to 128 in this study, and because multi-head attention requires the embedding dimension to be divisible by the number of heads, we choose four heads so that each head operates on a 32-dimensional subspace, providing a practical trade-off between modeling capacity and computational overhead. This interaction process helps capture the entity relationship between ships and EC nodes, thereby enhancing the model’s generalization ability across different scenarios. Because the final output is a global state embedding that is insensitive to the ordering of ships and EC nodes, the input is treated as an unordered set, thereby obviating the need for positional encoding. Subsequently, a global pooling operation is applied to the output of the Transformer encoder to compress the interactive information among entity tokens into a lower-dimensional, relational feature vector. Additionally, as channel occupancy conflicts are addressed during action selection, and channel IDs are included within the action tokens, the remaining global physical features are directly compressed into a dimension d via a two-layer multi-layer perceptron (MLP). Finally, the relational feature vectors from the Transformer encoder are added to the global physical feature vectors from the linear layers, and the final linear mapping is applied to generate the resultant state embedding representation:

**Table 1:** Three feature groups.

Group	Tensor Shape	Contents
Ship feature	(*M*,9)	position (*x*,*y*), task complexity, data size, local computing frequency, maximum power limit, energy coefficient, task arrival rate, latency tolerance
EC node feature	(*N*,6)	position (*x*,*y*), EC node computing frequency, max parallelism, previous utilization, previous mean load
Global rest feature	(*D*-9*M*-6*N*)	wavelength matrix, small-scale fading coefficient, per-link power


z=W(Mean(Transformer(relation))+MLP(global)). (24)

**Fig 4:**
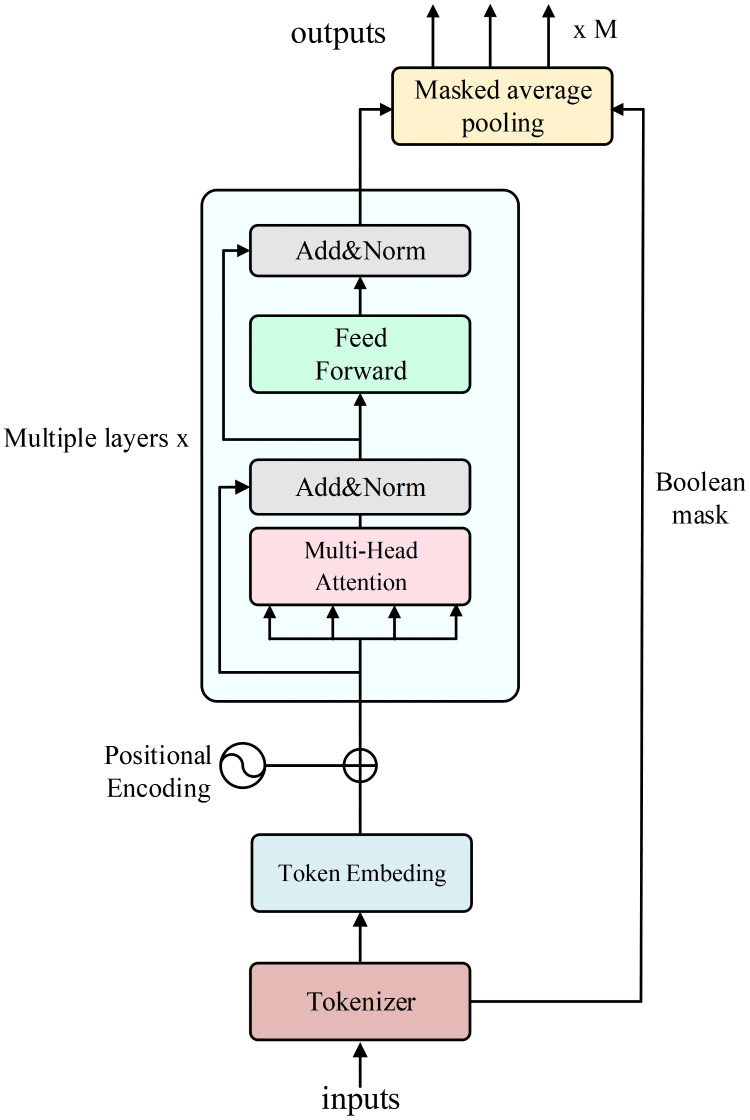
Architecture of the action encoder.


The architecture of the action encoder is illustrated in Fig 4. To effectively handle the high-dimensional, combinatorial action space within a DRL model and to support action factorization, we designed a dedicated tokenizer module within the action encoder. In the scenarios of this research, the decision-making of each ship involves both node selection and multichannel combinations. However, the traditional one-hot encoding method causes a dimension explosion. This may result in a large and sparse vector, which is not conducive to model’s learning and generalization. Specifically, if the one-hot encoding method is used, the dimension of the entire joint action space is:

$$d_{total\_onehot}=(1+N({\text{2}}^{K}-1){)}^{M}.$$ (25)

To address this problem, the tokenizer module adopts a sequential representation method based on tokens. Through a predefined vocabulary and conversion logic, this module converts the action, which is the offloading decision of each ship, including its ID, the target node ID (local or an EC node), and the selected channel IDs, into a fixed-length integer token subsequence. The subsequence is then padded using a special padding ID. The subsequences for all M ships are concatenated into a flattened sequence with a total length of M(2+K). Meanwhile, a Boolean mask is generated to identify the tokens in the sequence that belong to each ship decision. This serialization method significantly compresses the original action representation dimension. After that, the flattened token sequence is fed into the action encoder. It passes through a token embedding layer to map the discrete token IDs into feature vectors. Unlike the state encoder, the action encoder retains positional encoding, because the order of tokens in the action sequence contains important structural information—the relative positions of tokens that define the syntax of a single decision. In addition, the overall structure of the joint action is constituted by different ships’ decision sequences. Therefore, positional encoding is crucial to help the subsequent Transformer module understand these inherent sequential and structural dependencies. The feature sequence with positional information is then fed to a multilayer, four-head Transformer encoder. Similarly, the action Transformer uses an embedding dimension of 128 and employs four attention heads, with each head operating on a 32-dimensional subspace. This choice satisfies the divisibility requirement of multi-head attention and maintains a lightweight yet expressive encoder for the tokenized combinatorial actions. We adopt the same Transformer configuration in both the state and action encoders for architectural consistency. Through its self-attention mechanism, the encoder can effectively capture the complex dependencies in the sequence, generating deeply contextualized action representations. Finally, to achieve action factorization, the model utilizes a pre-generated Boolean mask to perform a masked average pooling operation on the output of the Transformer. This operation extracts a separate, fixed-dimension factorized action embedding vector for each ship. These factorized action embeddings are integrated with the global state embedding, and then are respectively fed into the corresponding ship advantage heads. As a result, the Q value of the combined action can be evaluated based on the marginal contribution of each ship.

#### 4.2.3 Rainbow DQN.

AF-TT-RDQN is built upon the Rainbow DQN. Rainbow DQN is an innovative extension of the original DQN algorithm, integrating six improvement mechanisms. These mechanisms are as follows:

1) Double DQN alleviates the overestimation problem of Q values in the original DQN. This is achieved by decoupling the process of action selection and value evaluation. Specifically, the evaluation network is first used to determine the optimal action, and then the target network is used to evaluate the value of that action.
2) Dueling DQN modifies the network architecture by decomposing the Q value into the state value function and the advantage function. This design promotes the model to obtain more detailed information, hence improving the stability and convergence speed of the training.
3) PER replaces uniform experience replay in the standard DQN. It assigns different priorities to experiences according to the magnitude of their Temporal Difference (TD) error. Therefore, the more valuable experiences are replayed frequently, which enhances the model’s learning efficiency.
4) Distributional DQN estimates the distribution of returns rather than calculating the expected value. It can provide a more detailed description of the uncertainty in values, avoid potential risks, and significantly enhance the network’s adaptability in handling complex tasks.
5) Multi-step learning introduces an N-step truncated reward update mechanism. It enables each update to gather more information from the environment, thereby effectively reducing the deviation in target value estimation at the beginning of training and further accelerating the entire training process by enhancing sample efficiency.
6) NoisyNet replaces the traditional ε-greedy exploration strategy by introducing learnable noise in the network parameter space. It encourages exploration more smoothly and improves the diversity of action selection.

The combination of these six mechanisms enables the Rainbow DQN to demonstrate performance significantly better than the traditional DQN across a wide range of reinforcement learning tasks [43–45].

**Fig 5:**
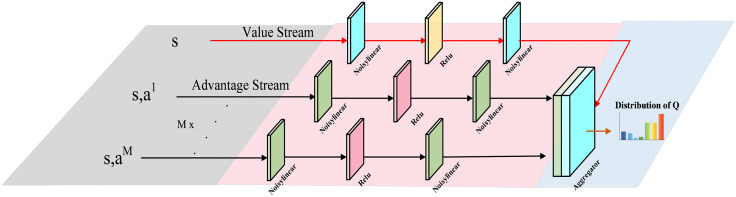
Architecture of evaluation and target networks.

Based on the Rainbow DQN framework, the evaluation network and target network, as shown in Fig 2, share the architecture illustrated in Fig 5. This architecture integrates multiple key techniques, including Dueling networks, NoisyNet, and distributional DQN, to accurately estimate the probability distribution of the state-action values. The network receives the state embedding, processed by the state encoder, and the independent action embedding for each ship, processed by the action encoder. These embeddings are then fed into the leading network. The state embedding enters the value stream, which consists of a series of NoisyLinear layers and ReLU activation functions, to output the distribution of the state value function. For each of the M subactions, the corresponding action embedding is concatenated with the state embedding. This concatenated vector is then passed to a dedicated advantage head, also composed of NoisyLinear layers and ReLU activation functions, to generate the advantage function distribution for each subaction. Finally, the network achieves the distribution of the Q value by summing and concentrating all streams.

#### 4.2.4 Parallel training mechanism.

**Fig 6:**
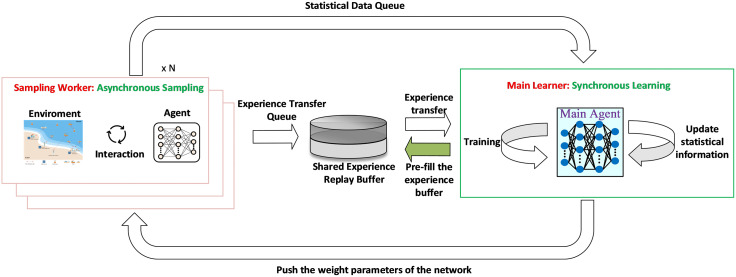
Parallel training framework based on asynchronous sampling and centralized synchronous learning.

To improve sample efficiency and stabilize training, recent reinforcement learning systems have widely adopted distributed parallel training frameworks. These frameworks typically employ multiple parallel worker agents to explore the environment and share a central network to update the parameters through synchronous or asynchronous mechanisms. Representative examples are A2C, Ape-X, IMPALA, R2D2, and DISTRL. In this study, the challenges in sampling efficiency and training stability come from the high complexity of the environment and the large volume of experience data. To address these problems, an asynchronous environment sampling and centralized synchronous learning-based parallel training framework is developed to improve policy updating stability and interaction efficiency. Our computation offloading algorithm is implemented and executed within this framework to leverage its scalable architecture and efficient learning processes fully. Fig 6 illustrates the parallel training framework.

As shown in Fig 6, multiple parallel sampling workers interact with independent environment replicas to collect state transition samples asynchronously, which are further transferred to the shared experience replay buffer through a respective experience transfer queue. Simultaneously, statistical data such as episodic rewards obtained by workers are transmitted to the main learner through a dedicated statistical data queue. It should be noted that agents in each worker process are used for environment interaction and data sampling without requiring backpropagation and network parameter updates. Each sampling worker operates independently, eliminating the need for mutual synchronization, which significantly improves sampling throughput and increases the speed of data collection, as well as the diversity of the collected experiences. At the initial stage of training, the main learner is capable of prefilling the shared experience replay buffer to guarantee the stability of training. After that, the main learner periodically samples mini-batches from the shared replay buffer to conduct network backpropagation and update its parameters. Furthermore, the main learner uses the latest weight overwrite strategy to broadcast updated network parameters to all sampling workers regularly. Consequently, each worker constantly applies the latest policy during environmental interaction and data collection. Centralized management of the shared experience replay buffer and unified parameter updates by the main learner ensure that the network parameters for every update are based on the latest global data. In this way, it effectively alleviates the parameter fluctuation problem caused by asynchronous updates, achieving significantly better stability and convergence during training. This framework effectively alleviates performance bottlenecks during the training process. It separates the data collection from the model training. In addition, this separated sampling and training design offers strong scalability and is convenient for future expansion to multi-GPU configurations or distributed, multi-node clusters.

#### 4.2.5 Overall operational flow.

The entire process can be divided into three main stages: initialization, parallel data collection, and centralized model training. Each stage is explained in detail as follows:

1) System Initialization and Setup: The system determines physical and operational parameters during the initialization phase. Then, the computing framework starts a centralized learning process (Main Learner) and multiple parallel sampling worker processes (Sampling Workers). The communication among these processes is achieved through dedicated message queues. In addition, the Main Learner initializes the comprehensive agent architecture. After all the initializations are complete, the Main Learner packages the initial weights of the evaluation network and broadcasts them to all the Sampling Workers.
2) Distributed Data Collection: Each Sampling Worker interacts with the environment to generate a series of comprehensive and diverse experience data for the Main Learner to use. The operation process is as follows. The Sampling Workers fetch the latest model parameters from the Main Learner and synchronize their local network configurations. At each beginning of the sampling episode, the workers collect the dynamic parameters of the environment corresponding to the current temporal step and integrate them with static parameters to construct high-dimensional, normalized state vectors. The workers process the state vectors through their local evaluation network to derive joint action vectors. After the joint operation is executed, the system calculates the resultant total latency and energy consumption metrics in compliance with the actions and the underlying physical model. Subsequently, reward signals are calculated with penalty terms for any constraint violations based on the normalized latency and energy consumption values. Then, the environment transitions into the subsequent temporal step. The workers fetch the updated dynamic parameters and construct the following state vectors. Every interaction experience is stored in the local N-step buffers maintained by the workers. If the buffers reach saturation or the tasks are completed, the workers calculate the cumulative rewards over N steps and the corresponding discount factors. The complete N-step experience data are then transmitted to the Main Learner through the established communication queues.
3) Centralized Model Training: To alleviate cold start problems, the Main Learner firstly fills up the PER buffer by generating random samples. Once the formal training processes start, the Main Learner retrieves the N-step experience data transmitted by the workers via the communication queue and pushes them into its PER buffer. When the data accumulated in the experience replay buffer exceeds the required threshold, the Main Learner initiates its iterative learning process by sampling mini-batch experiences from the PER buffer. As prioritized replay mechanisms have been implemented, the samples with higher TD error are more likely to be chosen. For each sample in the batch, both the current Q-value distribution and the target Q-value distribution are calculated. The cross-entropy loss is calculated using the current and target Q-value distributions. Subsequently, backpropagation is executed to update the parameters of the evaluation network. Meanwhile, samples in the PER buffer are updated according to the calculated TD errors. At predetermined time intervals after completing the specified number of training iterations, the Main Learner transfers the weights of the evaluation network into the target network, aiming to enhance stability during training. Additionally, the Main Learner periodically broadcasts the latest evaluation network weights to all Sampling Workers in order to ensure exploration by the most current policy parameters.

This end-to-end process, which concurrently handles time-consuming environmental interactions and employs an efficient centralized learning approach, has constructed a complete high-performance reinforcement learning system. It enables the model to make effective computing offloading strategies based on the environment, solving the complex decision-making problems discussed in this study.

## 5. Simulation and results

### 5.1 Simulation setup

The effectiveness of computation offloading strategies learned by AF-TT-RDQN was evaluated through simulation-based comparisons with several baseline methods from the perspective of the weighted sum of the latency and energy consumption. The experimental environment consists of servers equipped with NVIDIA GeForce RTX 3090 graphics cards. All simulations were conducted using Python 3.12 and PyTorch 2.3.0 on the Ubuntu 22.04 operating system. Additionally, CUDA 12.1 is utilized for GPU acceleration.

We constructed a near-shore grid area measuring 10 × 5 km. Considering the heterogeneity of ships and EC nodes, we deployed five types of ships positioned at coordinates (−2.5, 2.8), (0.5, 0.8), (1.5, 2.5), (3.5, 2.0), and (4.5, 1.5) km, and three types of EC nodes located at (0.0, 0.0), (3.0, 0.5), and (−3.0, 2.0) km. The maximum number of uplink channels from each ship to each EC node was set to two. The other parameters used in the simulations are listed in Table 2. The task arrival rate of each ship and the maximum allowable latency are generated using a predefined function at each decision slot.

**Table 2:** Parameters used in the simulation.

Notation	Description	Values
$c_{i}$	Number of CPU cycles required to execute each computation task on ship i	[220, 420] *M-cycles*
$f_{i}$	Local computing frequency of the ship i	[250, 500] *MHz*
$b_{i}$	Input data size of each computation task on the ship i	[2, 6] *MB*
$z_{i}$	Ship-specific energy coefficient	[6.0 × 10^-28^, 4.0 × 10^-27^] *Js*^*2*^*/cycle*^*2*^
$\overset{max}{\underset{i}{P}}$	The maximum power limit of the ship’s power	[7, 17] *W*
$\overset{k}{\underset{i,j}{p}}$	Transmission power of the ship i on the channel to the EC node j	[1, 6] *W*
W	Bandwidth of each subchannel	10 *MHz*
$\sigma^{\text{2}}$	Noise power	−100 *dBm*
$\lambda_{j,k}$	Wavelength of the k th subchannel of EC node j	[0.0852, 0.6522] *m*
$K_{r}$	Rician factor	5
$f_{j}$	Computing frequency of EC node j	[8.5, 30] *GHz*
$\overset{max}{\underset{j}{N}}$	Limitation on the number of processing threads at the EC node j	[4, 20]
α	Weight parameter for latency	0.5
β	Weight parameter for energy consumption	0.5

To evaluate the effectiveness of computation offloading strategies learned by AF-TT-RDQN, the following baseline schemes are used for comparison:

- Local Execution: All tasks are executed locally on ships, eliminating the need for offloading.
- Complete Edge Execution: All tasks are offloaded to EC nodes for processing.
- Random Execution: Each task is randomly selected to be either processed locally or offloaded to EC nodes.
- Discrete Particle Swarm Optimization (DPSO) [46]: A meta-heuristic optimization algorithm adapted to handle discrete computation offloading decisions.
- Advantage Actor-Critic (A2C): A synchronous policy gradient reinforcement learning method utilizing advantage estimation to improve training stability.
- Trust Region Policy Optimization (TRPO): A policy-gradient reinforcement learning algorithm that guarantees monotonic improvement through constrained updates within a trust region.
- Proximal Policy Optimization (PPO): An efficient variant of TRPO that simplifies the policy update mechanism through a clipped surrogate objective while retaining comparable performance.

Specific network parameters of AF-TT-RDQN are described in Table 3.

**Table 3:** Network parameters of AF-TT-RDQN.

Notation	Description	Values
$N_{atoms}$	Number of atoms for distributional DQN	51
$V_{min}$	Minimum value of support	−1200.0
$V_{max}$	Maximum value of support	1.0
γ	Discount factor	0.98
χ	Learning rate	4 × 10^−4^
B	Batch size	128
C	Replay memory capacity	50,000
$N_{step}$	*N*-step return	3
$\alpha_{PER}$	Prioritized replay exponent	0.5
$\beta_{\text{0}}$	Initial importance sampling weight	0.4
$\beta_{frames}$	Frames for importance sampling annealing	20,000
$\sigma_{\text{0}}$	Initial noise parameter for NoisyNet	0.7
$N_{workers}$	Number of parallel workers	5

### 5.2 Training results of the proposed algorithm

First, we conduct experiments to evaluate the training convergence of AF-TT-RDQN. In each training episode, the algorithm interacts with the environment for 500 steps.

**Fig 7:**
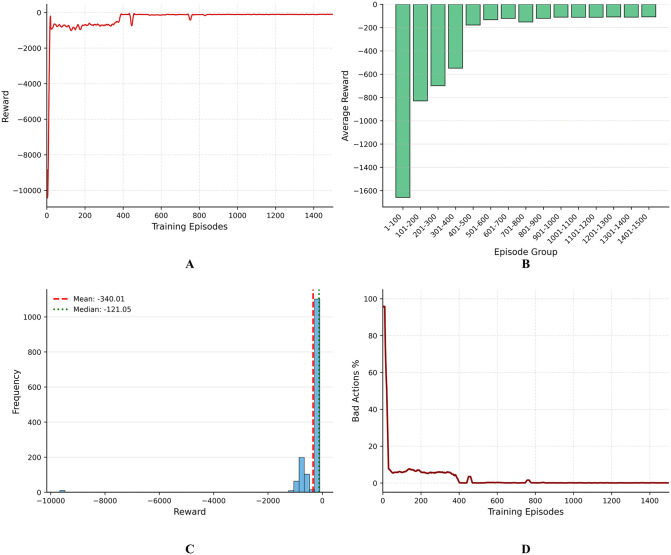
Training results of AF-TT-RDQN from multiple perspectives. A: Episode reward convergence. B: Average reward per 100-episode intervals. C: Frequency distribution of episode rewards. D: Bad action percentage over episodes.

Fig 7 shows the training results of AF-TT-RDQN from multiple perspectives. Fig 7A illustrates the reward convergence curve over 1500 training episodes. Initially, the reward displays a considerable negative value (approximately −10,000), indicating frequent constraint violations and inefficient initial offloading strategies. As training proceeds, the reward rises rapidly. It stabilizes in the range of −500–0, proving that the proposed algorithm can learn efficient and constraint-compliant computation offloading strategies effectively over time. The reward curve indicates that the algorithm achieves stable convergence after 750 episodes, and the subsequent training stage exhibits minimal fluctuations, indicating that its strategy learning effect is highly robust. Fig 7B presents a detailed analysis of the comprehensive performance, illustrating the learning progress between different training stages. By calculating the average reward over consecutive 100-episode intervals, it can be observed that there has been a significant improvement. It rises from around −1600 points at the first stage (episodes 1–100) to roughly −100 points at the final stage (episodes 1401–1500). The dramatic performance improvement occurs between episodes 101 and 400, where the average reward increased by more than 1000 points. This indicates that the algorithm effectively balances exploration and exploitation during the early learning stage. It further demonstrates that the optimization of learned strategies effectively reduces latency and energy consumption, as intended by the reward design. The reward distribution in Fig 7C reveals the important characteristics of the learned strategies. Although the average reward is −340.01, the median value of −121.05 suggests a right-skewed distribution, with a concentration of episodes achieving rewards closer to −100. The histogram is bimodal, with a dominant peak near −100 and a secondary peak near −500, indicating occasional returns to suboptimal solutions while maintaining a good overall performance. Fig 7D illustrates the trend of the percentage of bad actions, defined as actions that violate system constraints during the training episodes. It starts at almost 100% at the beginning and reaches below 10% within the first 200 episodes, then remains at a level close to 0% after 750 episodes. This rapid reduction in bad actions demonstrates that the algorithm can quickly identify and avoid harmful behaviors, which is crucial for the safe and efficient implementation of strategies.

**Fig 8:**
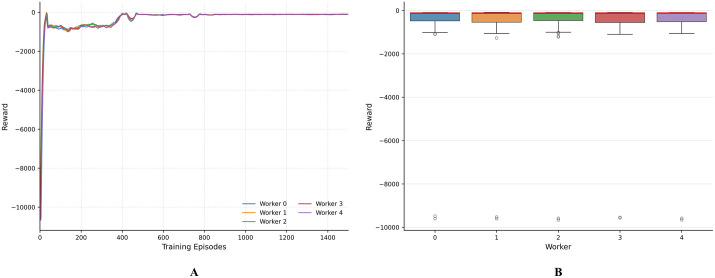
Parallel worker performance analysis in AF-TT-RDQN. A: Individual worker reward convergence. B: Reward distribution by the worker.

Fig 8 presents an analysis of the parallel workers’ training performance in AF-TT-RDQN. The results show that although the five parallel workers explored separately, their outcomes are highly consistent. Fig 8A illustrates that the individual worker reward convergence curves exhibit highly synchronized learning patterns throughout the 1500-episode training period. All five workers exhibit similar exploration behavior at the beginning, with rewards starting around −10000 and improving rapidly during the first 200 episodes. The convergence curves indicate a slight difference among the workers, and all curves stabilize between −500 and 0 around episode 400. This level of synchronization is notable, as each worker interacts with their own environment, and there is no direct coordination between them. These results suggest that centralized parameter updates can help maintain strategy consistency across a distributed system. The reward distribution analysis in Fig 8B provides quantitative evidence of consistency in performance among workers. The box plots reveal that all workers achieve comparable median rewards. Worker 1 has a slightly higher median (approximately −100), while the others range from −100 to −400. The interquartile ranges are relatively consistent across all workers, indicating similar performance stability. Outliers are evenly distributed, with each worker occasionally receiving very low rewards. This is expected because of the randomness of the environment and the need for exploration.

### 5.3 Training convergence comparison

**Fig 9:**
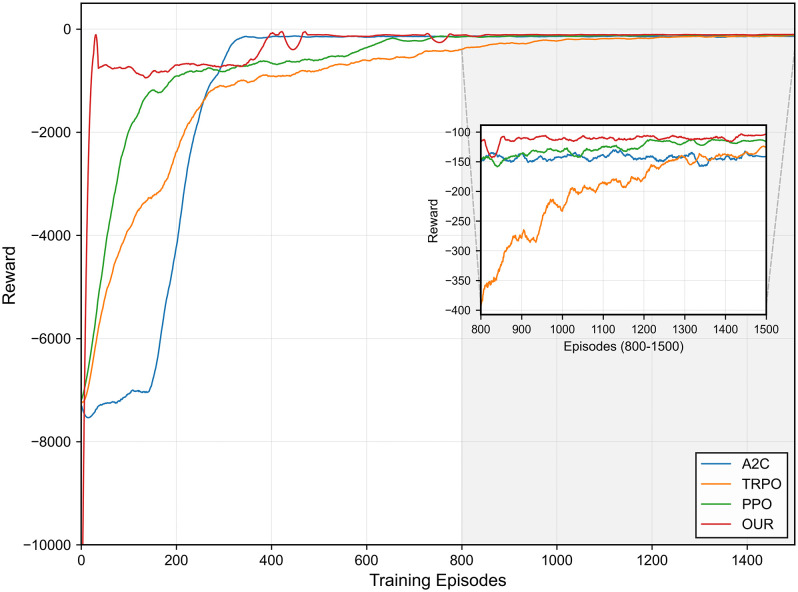
Training convergence comparison of AF-TT-RDQN against baseline DRL methods.

Fig 9 illustrates the comparison of the training convergence between our proposed algorithm and three baseline DRL methods: A2C, TRPO, and PPO. The proposed algorithm is an improvement based on the value-based algorithm. The specific details of the direct improvement compared with the related pioneering algorithms are elaborated in the subsequent ablation study. Here, we specifically select these prominent policy-based algorithms as baselines because they are widely recognized for their strong performance and stability in dealing with problems involving the high-dimensional discrete action space, which is the focus of this study. The results demonstrate the excellent convergence performance of our approach across the 1500-episode training horizon. Consistent with prior experiments, each training episode involves 500 steps of interaction with the environment. As shown in Fig 9, all algorithms exhibit a clear upward learning trend, with the average reward increasing gradually as training progresses. However, significant differences in the convergence speed, stability, and final converged performance are observable. During the initial training phase (approximately episodes 0−300), AF-TT-RDQN, PPO follows, exhibits the fastest learning speed and achieves high reward values significantly earlier than TRPO and A2C. This indicates that AF-TT-RDQN has high sample efficiency and can find effective strategies more quickly. During the training process for episodes 300–800, the proposed algorithm continues to demonstrate excellent convergence speed and performance despite minor fluctuations. During this same period, A2C clearly experiences a significant boost in its convergence speed, surpassing that of the other methods in finding a strong solution relatively early. This can be attributed to the fact that the A2C algorithm does not have strict limitations on the step sizes for policy updates, allowing it to explore more aggressively. In the early stages of training or during certain phases, if the environment provides clear and strong reward signals, A2C can leverage these signals to make substantial policy updates, thereby rapidly improving its performance. By contrast, PPO and TRPO converge more slowly than the other DRL algorithms due to the intrinsic mechanism that limits the change in the policy. The purpose of the constraints is to stabilize the updates, prevent new policies from being significantly different from previous ones, and allow for more gradual and smoother improvements. This behavior reflects the fundamental trade-off between the convergence speed and stability among the different DRL algorithms. The inset plot provides detailed information about asymptotic performance during episodes 800−1500. Our proposed algorithm performs best and stabilizes, achieving the highest average reward of −100 with minimal variance. PPO has the second-best performance, with rewards stabilizing near −120. TRPO converges more slowly and ultimately stabilizes at a slightly higher level than −150. Although A2C achieves early convergence, its final performance remains relatively low. Overall, the proposed algorithm achieves a good balance between the speed of convergence, training stability, and final convergence performance.

### 5.4 Optimization performance comparison

**Fig 10:**
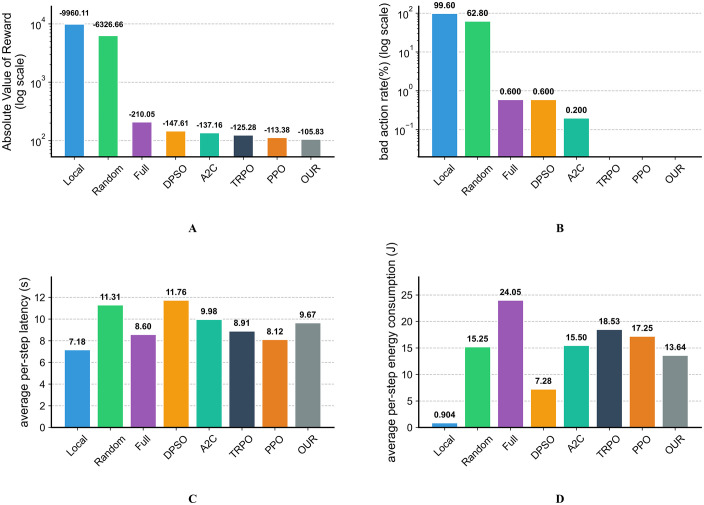
Optimization performance comparison of AF-TT-RDQN against baseline methods. A: Comparison of total reward. B: Comparison of bad actions. C: Comparison of average per-step latency. D: Comparison of average per-step energy consumption.

To evaluate the effectiveness of computation offloading strategies learned by AF-TT-RDQN, we compare the optimization performance with that of several baseline methods across four key metrics. The baselines include DRL algorithms (A2C, TRPO, and PPO) and non-learning-based methods (Full Local Execution, Full Edge Execution, Random Execution, and DPSO). For fairness, all methods are tested under the same simulation parameters. In particular, the parameters of DPSO are carefully tuned to match the execution time of the proposed algorithm, enabling a comparison under similar computational complexity. Each method interacts with the environment for 500 steps, and the results are shown in Fig 10.

Fig 10A compares the total reward, which is the primary evaluation metric combining the weighted sum of the latency and energy consumption, with penalties for constraint violations. The proposed algorithm achieves a total reward of −105.83, which is significantly better than all the baseline methods. It outperforms PPO by 6.7%, TRPO by 15.5%, and A2C by 22.8%. This result demonstrates that our algorithm effectively balances the conflicting objectives. DRL-based methods (PPO, TRPO, A2C) also outperform the heuristic DPSO and the simple strategies (“Local,” “Random,” “Full”). The extremely low rewards for “Local” (−9960.11) and “Random” (−6326.66) reflect their inability to handle the system’s complex constraints. These methods incur large penalties due to frequent violations, confirming the need for an intelligent decision-making mechanism. Fig 10B illustrates the bad action rate, defined as the percentage of actions that violate the system constraints. AF-TT-RDQN, together with TRPO and PPO, achieves a 0% bad action rate, indicating that the learned strategies fully meet all operational limits, including maximum latency tolerance and power constraints. This demonstrates the robustness and reliability of DRL-based methods for learning safe and practical offloading strategies. In contrast, the “Local” and “Random” strategies have very high bad action rates (99.6% and 62.8%, respectively), making them unsuitable for real-world use. Figs 10C and 10D illustrate the average per-step latency and energy consumption, considering only valid actions. This distinction is important for a fair and meaningful interpretation of the results. For example, the “Local” strategy appears to have the lowest latency (7.18 s) and energy consumption (0.904 J). However, as shown in Fig 10B, it suffers from a bad action rate of 99.6%, meaning that these values are derived from only 0.4% of cases where local execution is possible, and therefore do not accurately reflect its actual performance. A meaningful comparison of latency and energy consumption is most appropriate among algorithms that consistently produce valid actions. As a result, we mainly compare the reliable methods with a 0% bad action rate (OUR, PPO, and TRPO). Figs 10C and 10D are provided mainly for auxiliary analysis, while our primary objective remains minimizing the weighted sum of latency and energy consumption shown in Fig 10A. Therefore, although the proposed algorithm’s average latency (9.67 s) is slightly higher than that of PPO (8.12 s) and TRPO (8.91 s), it achieves a significantly lower average energy consumption (13.64 J) than PPO (17.25 J) and TRPO (18.53 J). Specifically, the latency of PPO is reduced by 16%, but its energy consumption is 26.5% higher than that of our algorithm. The latency of TRPO is decreased by 7.9%, but its energy consumption is increased by 35.9%. This ensures that the overall total reward of our proposed algorithm remains the highest in the simulation, indicating that it learns more efficient computation offloading strategies than the other baselines.

### 5.5 Ablation study

To evaluate the impact of each key component on AF-TT-RDQN, we conduct an ablation study. We systematically remove or replace five important modules: the Transformer in the state encoder, the Transformer in the action encoder, PER, distributional DQN, and multi-step learning, thereby generating corresponding variants. Through the experiment, we compared the performance of each variant with that of AF-TT-RDQN. All the experimental results presented in Table 4 are cumulative values obtained after interacting with the environment for 500 steps.

**Table 4:** Ablation study of key components in AF-TT-RDQN.

Method	State Transformer	Action Transformer	PER	Distributional DQN	Multi-step learning	Total reward	Bad action rate
1	×	√	√	√	√	−126.03	0.2%
2	√	×	√	√	√	−637.03	5.2%
3	√	√	×	√	√	−275.31	0.8%
4	√	√	√	×	√	−207.17	0.4%
5	√	√	√	√	×	−127.59	0.2%
6	√	√	√	√	√	−105.83	0%

As shown in Table 4, AF-TT-RDQN (Method 6) yields the best results in terms of both total reward (−105.83) and bad action rate (0%), indicating that the integration of all components has a positive impact. Among these components, the Transformer in the action encoder exhibits the most prominent influence. According to Method 2, replacing the Transformer in the action encoder with a standard MLP results in a sharp decline in total reward to −637.03 and an increase in bad action rate to 5.2%. This shows that sequential modeling and the self-attention mechanism are crucial for capturing the complex structure of the high-dimensional, combinatorial action space in this study. In addition, removing PER (Method 3) and distributional DQN (Method 4) also leads to noticeable performance degradation, with total rewards falling to −275.31 and −207.17, respectively. This confirms that PER enhances learning efficiency by prioritizing high-value samples, while distributional DQN strengthens the robustness of the policy by modeling the return distribution. Removing the Transformer in the state encoder (Method 1) or multi-step learning (Method 5) also results in certain performance losses, with total rewards of −126.03 and −127.59, respectively. This suggests that the Transformer in the state encoder helps capture complex entity relationships from high-dimensional states. At the same time, multi-step learning accelerates model convergence and mitigates estimation bias by incorporating longer-term return signals. Overall, this ablation study strongly demonstrates that each integrated component makes a positive contribution to the algorithm.

## 6. Conclusion

To address the computation offloading problem in dynamic EC-IoS scenarios characterized by the high-dimensional, combinatorial decision space, strict system constraints, and rapidly varying maritime wireless channels, this study proposes AF-TT-RDQN. This algorithm can find the optimal strategies within the system that minimize the weighted sum of delay and energy consumption, while satisfying the system constraints. Extensive simulation results illustrate that the proposed algorithm outperforms several baselines. In the independent analysis of training convergence of AF-TT-RDQN, the results show that the algorithm can learn efficient computation offloading strategies that comply with the constraints, demonstrating strong robustness. Furthermore, the results show that although the five parallel workers of the algorithm operate independently, their training outcomes are highly consistent. In the training convergence comparison experiment, AF-TT-RDQN outperforms PPO, TRPO, and A2C in terms of training stability and final convergence performance. Most importantly, in the optimization performance evaluation comparison experiment, the offloading strategies learned by AF-TT-RDQN achieve the best total reward of −105.83, which is 6.7%, 15.5%, and 22.8% higher than those of PPO, TRPO, and A2C, respectively. Meanwhile, the strategies strictly adhere to all system constraints, achieving a zero rate of invalid actions, which is a necessary condition for practical applications. Moreover, the strategies learned by AF-TT-RDQN provide a more favorable latency–energy balance under the weighted-sum objective and achieve the highest total reward. Although the computation offloading latency produced by TRPO and PPO is 7.9% and 16% less than that of our algorithm respectively, the corresponding energy consumption increases by 35.9% and 26.5%, resulting in a lower total reward than ours. Additionally, the ablation studies confirm that each integrated component in the AF-TT-RDQN has made a positive and significant contribution to the algorithm’s final performance.

Despite specific achievements, the research also has certain limitations. To maintain tractability in a near-shore setting, we assumed stationary EC nodes and quasi-static ship locations, thereby ignoring explicit ship mobility and the resulting time-varying link distances. Future work will incorporate mobility-aware modeling, such as stochastic or trajectory-based mobility models, and investigate mobility-robust offloading strategies. In addition, in the problem definition, we adopted the M/M/1 queuing model to simplify the calculation of the task waiting times. Although this approach avoids the problem of state space explosion and enables the algorithm to focus on optimizing the system’s average performance, it sacrifices a certain degree of model accuracy. In future work, we will combine Lyapunov optimization to better reflect the real-time dynamics of the queue. Moreover, we aim to extend the framework to the joint optimization of discrete computation offloading decisions and continuous resource allocation variables, such as transmit power control and computational frequency scaling. Developing the algorithm capable of handling the mixed discrete-continuous action space would represent a substantial advancement in this field.
